# Disrupted Auto-Activation, Dysexecutive and Confabulating Syndrome Following Bilateral Thalamic and Right Putaminal Stroke

**DOI:** 10.1155/2008/693671

**Published:** 2008-07-15

**Authors:** Lieve De Witte, Sebastiaan Engelborghs, Jo Verhoeven, Peter P. De Deyn, Peter Mariën

**Affiliations:** ^1^Department of LinguisticsVrije Universiteit BrusselBrusselsBelgium; ^2^Department of NeurologyZNA Middelheim HospitalAntwerp and Laboratory of Neurochemistry and BehaviourInstitute Born-BungeUniversity of AntwerpAntwerpBelgium; ^3^Department of Health Care SciencesUniversity College AntwerpAntwerpBelgium; ^4^Department of Nursing SciencesFaculty of MedicineUniversity of AntwerpAntwerpBelgium; ^5^Department of Language and Communication SciencesCity UniversityLondonUK

**Keywords:** Putamen, bithalamic stroke, confabulatory syndrome, dysexecutive syndrome, psychic akinesia

## Abstract

Objective: Clinical, neuropsychological, structural and functional neuroimaging results are reported in a patient who developed a unique combination of symptoms after a bi-thalamic and right putaminal stroke. The symptoms consisted of dysexecutive disturbances associated with confabulating behavior and auto-activation deficits. Background: Basal ganglia and thalamic lesions may result in a variety of motor, sensory, neuropsychological and behavioral syndromes. However, the combination of a dysexecutive syndrome complicated at the behavioral level with an auto-activation and confabulatory syndrome has never been reported. Methods: Besides clinical and neuroradiological investigations, an extensive set of standardized neuropsychological tests was carried out. Results: In the post-acute phase of the stroke, a dysexecutive syndrome was found in association with confabulating behavior and auto-activation deficits. MRI showed focal destruction of both thalami and the right putamen. Quantified ECD SPECT revealed bilateral hypoperfusions in the basal ganglia and thalamus but no perfusion deficits were found at the cortical level. Conclusion: The combination of disrupted auto-activation, dysexecutive and confabulating syndrome in a single patient following isolated subcortical damage renders this case exceptional. Although these findings do not reveal a functional disruption of the striato-ventral pallidal-thalamic-frontomesial limbic circuitry, they add to the understanding of the functional role of the basal ganglia in cognitive and behavioral syndromes.

## 1. Introduction

Ischemic lesions of the basal ganglia and the thalamus may result in clinical syndromes characterized by a wide range of symptoms varying from pure motor deficits to complex sensory, neuropsychological and behavioral alterations [5,52,67]. There is ample evidence that ischemic lesions of the basal ganglia result in motor and neurobehavioral syndromes in which dystonia and apathy are common [6,14,21,28,32,52,54, 71]. Bilateral vascular thalamic lesions may induce a wide range of neurobehavioral symptoms such as: 1) transient vigilance disorders, 2) hypersomnia, 3) verti-

ISSN 0953-4180/08/$17.00 © 2008 – IOS Press and the authors. All rights reserved cal gaze palsy, 4) anterograde amnesia, 5) speech and language disturbances and 6) apathy [3,20,24,30,31, 35,38,49,50,57,59,61,73]. A typical syndrome of apathy which has been documented in association with uni- or bilateral lesions of the thalamus and/or the basal ganglia [10–12,14,15,19,23,31,68,70,72] is known as 'athymhormia', 'psychic akinesia', or 'loss of psychic self-activation' (LPSA), of which two distinct subtypes can be distinguished. The first variant is a condition of extreme loss of activity and motivation resulting in apathy, motor and verbal aspontaneity with subjacent 'mental emptiness'. Remarkable in this condition is the sharp contrast between impaired self-activation and intact heteroactivation of behavior. The second behavioral condition consists of an apparent flatness or poor expressiveness of affect and the disappearance of psychic life [11,13,23,39,42,45].

Levy and Dubois [62] define apathy as an 'observable pathologic behavioral syndrome consisting of a quantitative reduction of self-generated voluntary and purposeful behaviors' and distinguish three subtypes in the underlying mechanisms of apathy: 'emotional-affective', 'cognitive' and 'auto-activation'. Apathy related to a disruption of 'emotional-affective' processing refers to a reduction of goal-directed behavior due to an inability to associate affective and emotional signals with ongoing and forthcoming behaviors, whereas apathy related to a disruption of 'cognitive' processing is related to cognitive deficits in elaborating plans of action. The third and most severe form of apathy consists of a disruption of auto-activation processing which refers to the inability of self-activating and self-initiating thoughts and activities: this affects both cognitive and emotional responses. This behavioral syndrome is similar to 'athymhormia', 'psychic akinesia' and 'loss of psychic self-activation'. The 'auto-activation' deficit mostly involves bilateral lesions of the thalamus, the pallidal globe, the caudate nucleus or the striato-pallidal region. Although the condition most commonly occurs after bilateral lesions, patients with unilateral lesions have been reported [1,4,8,11,23, 26,29,31,33,37,45,46,51,53,65,66,68,69,71]. Besides psychic akinesia, impairments in executive functioning and confabulations are also associated with bi-thalamic lesions [20,22,38,40,70,73] .

This article reports the clinical, neuropsychological, structural and functional neuroimaging results in a patient who developed a unique set of symptoms consisting of a dysexecutive syndrome in association with confabulating behavior and auto-activation deficits after a bi-thalamic and right putaminal stroke.

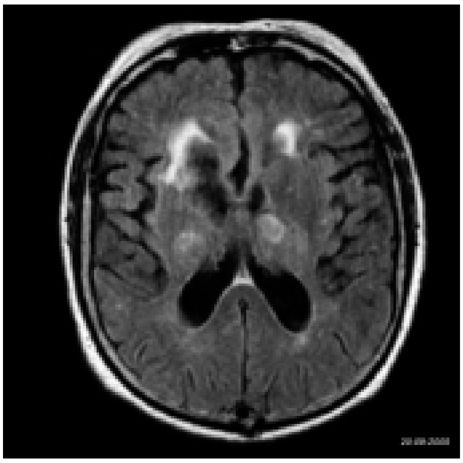

Fig. 1. Brain MRI axial FLAIR carried out five days after stroke revealing focal ischemic damage to both thalami and the right putamen.

## 2. Case report

A 78-year-old right-handed woman was admitted to hospital after a sudden onset of incoherent speech and confusion. Medical history was unremarkable. Apart from mild disorientation, clinical neurological examination on admission was normal. Tests of eye movements and other cranial nerve functions were normal. Brain magnetic resonance imaging (MRI) in the acute phase (5 days post-onset) revealed ischemic lesions in both thalami and the right putamen (Fig. 1). Comparison of the MRI-images with an atlas of neuro-imaging and neuroanatomy [27] showed unilateral involvement of the left medial and anterior thalamic nucleus, unilateral involvement of the right dorsal anterior nucleus, bilateral involvement of the intralaminar nuclei, unilateral involvement of the right putamen and bilateral involvement of the frontal subcortical white matter.

In the post-acute phase of the stroke (2 weeks post-onset), anomic language disturbances, concentration and memory deficits were found in combination with a dysexecutive syndrome. At the behavioral level, a combination of inhibitory and disinhibitory symptoms were found. Seven months post-onset, repeat neurolinguistic and neuropsychological investigations revealed severe frontal dysexecutive dysfunctions in combination with a unique neurobehavioral syndrome characterized by a severe loss of psychic self-activation and the presence of a confabulatory syndrome. $99^{m}$Tc ECD single photon emission computed tomography (SPECT) scan was carried out eight months post-onset. Trans-axial images with a pixel size of 3.56 mm were anatomically standardized using SPM and compared to a stan-

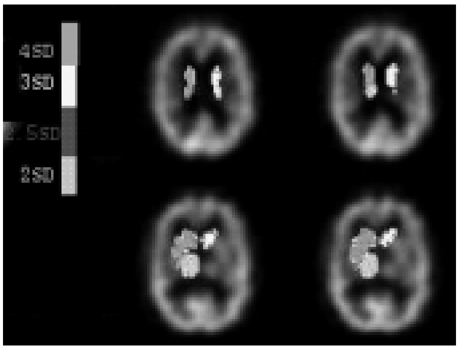

Fig. 2. Quantified ECD SPECT eight months after stroke disclosing hypoperfusions in the thalamus, both caudate nuclei and the left lentiform nucleus.

dard normal and SD image obtained from 15 normal ECD perfusion studies. Using a 31 ROI template the z-scores (SD) were then calculated for each region. A regional z-score of $> 2.0$ was considered significant. In comparison to normal database findings the quantified baseline ECD SPECT at eight months post-stroke showed a significant decrease of perfusion in both thalami (left: $-1.96$ SD; right: $-2.47$ SD), both caudate nuclei (left: $-5.24$ SD; right: $-3.92$ SD) and the left lentiform nucleus ($-4.97$ SD). No deficits were found at the cortical level (Fig. 2).

## 3. Methods

Formal neuropsychological investigations were carried out two weeks (acute phase) and seven months (late phase) after the stroke. Language was assessed by means of subtests of the Boston Diagnostic Aphasia Examination (BDAE) [25], subtests of the Aachen Aphasia Test (AAT) [56], the Boston Naming Test (BNT) [18,58], subtests of the Hierarchic Dementia Scale (HDS) [41] and a semantic verbal fluency task (unpublished norms). Cognitive functions were examined by means of the Mini Mental State Examination (MMSE) [43], the Colored Progressive Matrices (CPM) [35], the Hierarchic Dementia Scale (HDS) [41], the Trail Making Test (TMT) [63,64], the Stroop Color Word Test (SCWT) [36] and the Wisconsin Card Sorting Test (WCST) [7].

## 4. Results

Neurolinguistic and neurocognitive test results are summarized in Table 1. In the acute phase of the stroke, the patient obtained normal scores for auditory comprehension, written comprehension, repetition, reading and writing. Similar results were obtained in the late phase of the stroke. Two weeks post-onset, the visual confrontation naming subtest of the HDS (4/10) and the BNT (22/60; $-3.91$ SD) showed severe anomia. Most naming errors were semantic paraphasias (14/38) (e.g. tree for cactus), followed by perseverations (9/38), semantic neologisms (7/38) (e.g. "freezing pencil" for igloo), inadequate circumlocutions (4/38) (e.g. parts of a throw for domino) and visual errors (4/38) (e.g. cup for mask). Seven months post-onset a remission of naming errors was found (HDS: 10/10; BNT: 51/60 ($+1.41$ SD)). In a semantic verbal fluency task the patient was asked to name as many animals, means of transport, vegetables and clothes as possible within one minute for each semantic field. In the acute phase the patient produced 22 items ($-1.82$ SD). Her performance had improved considerably in the late phase (39 items; $-0.37$ SD). In the acute phase of the stroke spontaneous speech was characterized by incidences of neologistic and semantic jargon. Seven months later jargon had resolved completely. Despite normal oral language skills, the patient did not take any initiative to speak spontaneously.

At a neurocognitive level, the MMSE revealed a deficient score of 22/30 ($-3.33$ SD) two weeks post-onset neurological symptoms. A normalization of the total score was found seven months post-onset (26/30; $-0.66$ SD) [60]. As shown by the HDS subtest results, recent memory and concentration were disrupted in the acute phase. Seven months later, recent memory defects and concentration disturbances had resolved. Praxis, orientation, visual perception, gnosis and arithmetics were normal as assessed by the HDS. On the CPM, the patient obtained normal results in the acute and late phase of the stroke. Executive dysfunctions were found at both the psychometric and behavioral level. As demonstrated by a severely deficient score on the Stroop Color-Word Test (card III $=$ percentile 1) the ability of resistance to interference in more automatic stimuli was severely disturbed in the acute phase. Seven months later performance had improved (card III $=$ percentile 30). A severely defective result on the WCST (zero categories in 64 trials) indicated that mental flexibility, abstract reasoning, frontal planning and organization were severely impaired in both the acute and lesion phase of the stroke. Disturbed mental flexibility was also evidenced by a defective result on the TMT (part B: percentile 10).

At the behavioral level a range of frontal-like disturbances were observed such as apathy, disconcern,

Table 1
Neuropsychological test results obtained two weeks and seven months post-onset

| Neuropsychological tests | Week 2 | Month 7 | Percentile 2/7 | Max | Mean | SD |
|---|---|---|---|---|---|---|
| *Mini Mental State Examination* | 22 | 26 | | 30 | 27 | 1.5 |
| *Intelligence* | | | | | | |
| **Raven Coloured Progressive Matrices** | 23 | 31 | 75/95 | 36 | | |
| *Memory* | | | | | | |
| **Hierarchic Dementia Scale (HDS)** | | | | | | |
| registration (item 8) | 8 | 10 | | 10 | 9.17 | 0.94 |
| recent memory (item 20) | 2 | 8 | | 10 | 8.51 | 1.64 |
| remote memory (item 17) | 10 | 10 | | 10 | 9.62 | 0.80 |
| *Language* | | | | | | |
| **Boston Diagnostic Aphasia Examination (BDAE)** | | | | | | |
| complex auditory language comprehension | 14 | 15 | 93/100 | 15 | | |
| **Akense Aphasia Test (AAT)** | | | | | | |
| repetition total | 150 | 150 | 100/100 | 150 | 144.1 | 8.07 |
| **Boston Naming Test (BNT)** | 22 | 51 | | 60 | 43.3 | 5.45 |
| **Hierarchic Dementia Scale (HDS)** | | | | | | |
| naming (item 6) | 4 | 10 | | 10 | 9.87 | 0.34 |
| comprehension (item 7) | 10 | 10 | | 10 | 9.85 | 0.42 |
| reading (item 10) | 10 | 10 | | 10 | 9.83 | 0.56 |
| writing (item 18) | 10 | 10 | | 10 | 9.06 | 1.26 |
| **Verbal fluency – semantic** | 22 | 39 | | | 43.4 | 11.76 |
| animals | 6 | 11 | | 1 min | | |
| vegetables | 5 | 12 | | 1 min | | |
| clothing | 5 | 10 | | 1 min | | |
| means of transport | 6 | 6 | | 1 min | | |
| *Concentration* | | | | | | |
| **Hierarchic Dementia Scale (HDS)** | | | | | | |
| concentration (item 13) | 7 | 10 | | 10 | 8.94 | 0.87 |
| *Frontal functions* | | | | | | |
| **Trail Making Test** | | | | | | |
| version A | – | 49 sec | –/80 | | | |
| version B | – | > 450 sec | –/< 10 | | | |
| **Stroop Color-Word Test** | | | | | | |
| card I | 46 sec | 36 sec | 60/95 | | | |
| card II | 106 sec | 55 sec | 3/75 | | | |
| card III | > 197 sec | 107 sec | 1/30 | | | |
| **Wisconsin Card Sorting Test (WCST)** | 0 | 0 | 0/0 | 64 | | |
| *Praxis – Orientation – Visual Perception & Gnosis Calculation* | | | | | | |
| ideomotor praxis (HDS, item 3) | 10 | 10 | | 10 | 9.45 | 0.80 |
| ideational praxis (HDS, item 5) | 10 | 10 | | 10 | 9.81 | 0.45 |
| construction (HDS, item 12) | 10 | 10 | | 10 | 8.33 | 1.88 |
| drawing (HDS, item 15) | 10 | 10 | | 10 | 8.72 | 0.97 |
| orientation (HDS, item 11) | 10 | 8 | | 10 | 9.45 | 1.02 |
| visual perception (HDS, item 4) | 10 | 10 | | 10 | 9.87 | 0.49 |
| gnosis (HDS, item 9) | 10 | 10 | | 10 | 9.47 | 0.50 |
| calculation (item 14) | 10 | 10 | | 10 | 8.83 | 1.26 |

Legend: max = maximumscore; SD = standard deviation; sec = seconds.

indifference, loss of interest, loss of critical attitude, poor motivation, flattened affect and abusive language (cursing). In addition the patient often confabulated. In spontaneous conversations, for example, she said that her husband, who died 18 years ago, did not accompany her because he dislikes hospitals. When she was asked to describe a script, such as a typical morning routine, a bizarre mix of confabulations disrupted the content of her speech. Some of these confabulations were based on true habits in the past while others were false beliefs. Although she was a resident in a nursing home, she claimed to wake up at six o'clock to prepare breakfast for both her children. She insisted that her son lives with her on the second floor of her house and that he leaves the house every morning at seven o'clock to catch the train to go to work. She further claimed that her daughter has a room on the first floor of her house and that she leaves at eight to work in a sheltered workshop (= true but 28 years ago). The reality is that both her son and daughter had left the house a long time ago. At the moment of the examination, she claimed that her husband stayed at home with her daughter (= false). When the examiner told the patient that she stayed in a nursing home, she said that her daughter lives with her husband now (= false).

The frontal-like inhibition disturbances and the confabulatory syndrome have remained unchanged. Her son considers the behavioral alterations as the most striking consequence of the stroke. Instead of the active, talkative woman she used to be before the stroke, she remains apathetic, passive and confabulatory. Because of these changes, she was admitted to a nursing home where she avoids contact with other residents. She hardly speaks and takes no initiative to communicate. Nevertheless, minimal levels of speech are punctuated with spontaneous and provoked confabulations. When no strong external stimulation is given, she does not do anything spontaneously. On the Middelheim Frontality Scale (MFS), she scored 7/10 [55]. The Beck Depression Inventory (BDI) did not show evidence in favor of a depressive syndrome [2].

## 5. Discussion

This paper reports a patient with a unique combination of cognitive and behavioral symptoms due to a bilateral thalamic and right putaminal stroke. The patient developed a dysexecutive syndrome that persisted during follow-up. In addition to impaired performance on frontal tasks evaluating mental flexibility, frontal planning and organization, loss of psychic self-activation and a confabulatory syndrome are found. Loss of psychic self-activation is characterized by apathy, loss of initiative, loss of concern, loss of critical attitude, motor and verbal aspontaneity, mental emptiness and flattened affect. This behavioral syndrome shares clear semiological similarities with syndromes caused by lesions of the dorso-medial prefrontal cortex and corresponds to the third subtype of the apathetic syndrome described by Stuss et al. [16] and Levy and Dubois [62] as a 'behavioral apathetic syndrome' and an 'auto-activation deficit'. The confabulatory syndrome in this patient consists of a mix of spontaneous as well as provoked confabulations. Most impressive are the spontaneous confabulations in which the patient shows a definite certainty about the veracity of her assertions. Most of these spontaneous confabulations are based on true events or on habits in the past while the provoked confabulations mainly reflect false beliefs. These findings provide additional evidence for a positive correlation between the degree of mental flexibility and the degree of spontaneous confabulation and are in agreement with the hypothesis of Nys et al. [22]. In this patient, a long-term coexistence of spontaneous confabulations and mental a-flexibility is observed as evidenced by the poor scores on the WCST and the TMT.

From an anatomo-clinical point of view, the persistence of executive dysfunctions may be attributed to the lesion in the medial thalamic nucleus, the intralaminar thalamic nuclei or any combination of these nuclei. Our findings are consistent with previous studies demonstrating an association between a lesion consisting of a combination of thalamic nuclei damage and a persistent dysexecutive syndrome [47–49,73]. Regarding the possible pathophysiological mechanisms underlying the neurobehavioral syndrome of psychic akinesia and/or confabulation, most research suggests disruption of the striato-ventral pallidal-thalamic-frontomesial limbic loop [9, 17,22,31,44,50,62,67]. Quantified SPECT in this patient does not support this view since no perfusion deficits were found in the anatomo-clinically suspected frontal brain regions. The lack of SPECT evidence for a functional disruption of this circuitry may indicate that bilateral involvement of the thalamus associated with putaminal damage is sufficient to induce the observed cognitive and behavioral symptoms. However, further research is necessary to clarify the role of combined lesions of the basal ganglia and thalami in dysexecutive, auto-activation and confabulatory syndromes.

## 6. Conclusion

A patient is reported who developed a persistent dysexecutive syndrome with confabulations and auto-activation deficits after ischemic damage to both thalami and the right putamen. There appears to be no SPECT evidence for a functional disruption of the striato-ventral pallidal-thalamic-frontomesial limbic loop. At the behavioral level the unique semiological association of the 'auto-activation deficit' and a confabulatory syndrome has – to the best of our knowledge – never been described before.

## Acknowledgements

We are grateful to Mrs. Inge Bats (Institute Born-Bunge, University of Antwerp) for the photographic work. This research was supported by the Research Foundation – Flanders (FWO–F). S.E. is a postdoctoral fellow of the FWO-F.
